# The maize *Ga1-s* allele confers protection against *ga1* pollen in popcorn and dent corn

**DOI:** 10.1038/s41598-022-25261-8

**Published:** 2022-12-02

**Authors:** Adrienne N. Moran Lauter, Jode W. Edwards, M. Paul Scott

**Affiliations:** Corn Insects and Crop Genetics Research Unit, USDA-ARS, Ames, IA 50011 USA

**Keywords:** Genetics, Plant sciences

## Abstract

Because corn pollen can be carried great distances by wind, maintaining genetic purity of corn grain is challenging. The challenge is substantially reduced in popcorn, which carries the *Ga1-s* allele preventing pollination by *ga1* plants, which include the vast majority of non-popcorn commercial maize varieties in the U.S.. *Ga1-s* can be transferred into dent corn but the effectiveness of the *Ga1-s* allele in popcorn and dent corn has never been compared, which is important because each are regulated differently regarding GMO contamination. We compared pollen exclusion of commercial popcorn hybrids, *Ga1-s* dent corn hybrids and normal dent corn hybrids for their ability to exclude *ga1* pollen using a sensitive field-based assay. While both popcorn and *Ga1-s* dent corn had significantly better pollen exclusion than normal dent corn, popcorn was significantly better than *Ga1*-s dent corn on average. Some *Ga1-s dent* hybrids excluded as well or better than some popcorn lines suggesting that identification of hybrids comparable to popcorn is possible. The information in this study will support revised gene purity regulations potentially decreasing costs and increasing genetic purity of organic corn.

## Introduction

Organic corn is a rapidly expanding market, with an increase of 124% in land area used to produce organic grain from 2008 to 2019. In spite of this growth, organic corn still makes up less than 1% of the land area planted to corn^1^. Production of organic corn in the US Corn belt is complicated by the need to maintain genetic purity of the crop. In order to produce grain, pollen produced by the male flower (the tassel) must land on the female flower (the silks) and fertilize the egg cells that line the corn cob. Viable pollen can travel kilometers on air currents^2^. If pollen from a GMO field fertilizes plants in an organic corn field, GMO contamination can result. The USDA National Organic Standards (7 CFR Part 205) prohibits the presence of GMOs in organic products. In addition, many export markets require corn that be GMO-free. In contrast, in 2020, 92% of the U.S. corn crop contains transgenes^3^.

Organic corn producers apply several management strategies to minimize GMO contamination, including delayed planting and isolation from GMO corn by physical distance, but these practices are inconvenient, inefficient, and potentially costly. Genetic solutions for reducing contamination by unwanted pollen have been proposed^4,5^ based on a trait called gametophytic incompatibility. Gametophytic incompatibility is conferred in maize by the genetic locus *Ga1* (gametophyte factor 1)^6^, The allele of the *Ga1* locus called *Ga1-s* can effectively prevent pollination by plants of the *ga1/ga1* genotype. Reciprocal crosses, with *ga1/ga1* plants as female and *Ga1-s/Ga1-s* plants as males are successful, as are self-pollinations of both genotypes. Therefore, plants with the *Ga1-s/Ga1-s* genotype produce grain normally by self-pollinations or by pollinations with neighboring *Ga1-s/Ga1-s* plants, but pollinations by *ga1/ga1* plants in nearby fields are greatly reduced in a *Ga1-s/Ga1-s* field. The vast majority of dent corn varieties have the *ga1/ga1* genotype. There are no reports of transgenic varieties carrying the *Ga1-s* allele or the *Ga1-m* allele, which, like *Ga1-s* can pollinate *Ga1-s* corn. The *Ga1-s* allele has long been used in the popcorn industry to prevent unwanted pollination by dent corn which severely reduces popcorn quality^7^. This trait is known in the popcorn industry as “dent sterility”. Hoegemeyer^8^ proposed to use the *Ga1* system for maintaining the genetic purity of organic corn by preventing unwanted pollinations by GMO corn. Both public^9,10^ and private breeders have transferred the *Ga1-s* allele from popcorn into dent corn in order to develop varieties with improved genetic purity for organic corn production.

Corn hybrids with the *Ga1-s/Ga1-s* genotype create a regulatory challenge with regards to requirements for genetic purity testing because current regulations are based either on popcorn that is assumed to contain the *Ga1-s* allele, or all other classes of corn that are assumed to not carry it. For example, the Non-GMO Project is the market-leading organization focused on labelling GMO-free products. They currently include “corn (except popcorn)” in their high-risk list, specifically excluding popcorn^11^, even though popcorn and dent corn are the same species and the difference in the genetic risk of GMO contamination is well established to be conferred by the *Ga1* locus. Dent corn varieties containing the *Ga1-s* allele are still assigned to the high-risk list in part because no data is available that compares the ability of *ga1* dent corn, popcorn, and *Ga1-s* dent corn to exclude undesirable pollen. The difference in risk classification triggers different regulatory approaches that vary greatly in cost between popcorn and all other corn products.

The degree to which *Ga1-s* corn lines can exclude unwanted pollen is complicated by genetic background and zygosity of the *Ga1-s* locus^9^. In addition, QTL for genetic modifiers have been shown to effect pollen exclusion in popcorn^12^. Researchers examined representatives from Supergold, Amber Pearl, and South American popcorn heterotic groups for the presence of genetic loci that alter the ability of *Ga1-s* to exclude *ga1/ga1* pollen. These loci are called “modifier genes” for the ability to modify the phenotype of the *Ga1-s* locus. Within their panel of 311 lines, the range of pollen exclusion was 0–100%, exemplifying the wide range of genetic background effects.

The goal of this work was to quantitatively evaluate and compare the degree of pollen exclusion conferred by the homozygous *Ga1-*s allele to popcorn and dent corn hybrids. Comparison of the degree of pollen exclusion will help to understand the regulation of pollen exclusion by the *Ga1* system and will provide much-needed information for establishment of a regulatory framework for dent corn carrying the *Ga1-s* allele.

## Materials and methods

### Corn lines and experimental design

Six yellow popcorn lines (*Ga1-s*/*Ga1-s*), and six yellow PuraMaize^®^ (*Ga1-s*/*Ga1-s)* hybrids were obtained from commercial sources. In addition, four yellow *ga1*/*ga1* experimental dent corn hybrids were used as non-excluding control hybrids in this study. In this way, three genetic classes were represented: Popcorn, PuraMaize and Dent corn. All hybrids were developed for use in the US corn belt and were well adapted to the experimental growing conditions. In addition, a yellow homozygous *Ga1-s* line in W22 (MGSC 401D) and the yellow *ga1/ga1* public inbred lines B73, Mo17 and Oh43 were used in various control pollinations. An in-house population *ga1/ga1* corn that produces blue kernels was used as a grain color marker for the *ga1/ga1* genotype. This work does not involve collection of wild plant or seed specimens and complies with the IUCN Policy Statement on Research Involving Species at Risk of Extinction and the Convention on the Trade in Endangered Species of Wild Fauna and Flora.

The 16 *Ga1-s* hybrids representing three genetic classes were planted in a randomized complete block design with two replications per year for two years, 2019 and 2020. Individual experimental units were single-row plots spaced 0.76 m apart, 5.3 m long, and contained approximately 25 plants. The *Ga1-s/Ga1-s* pollinators, *ga1/ga1* blue corn and control lines (*ga1/ga1*) were planted in nearby plots. All plots were managed according to recommended practices for dent corn production in the region.

### Molecular characterization of the *Ga1* locus in *Ga1-s* hybrids

Experimental material was genotyped and the *ZmPme3* gene was sequenced to determine the presence of the same *Ga1-s* allele. Genomic DNA was extracted, primers PME_A and PME_C^13^ were used to PCR amplify the *ZmPme3* gene and samples were sent to the Iowa State University DNA Facility for Sanger sequencing in both directions. These amplify 1322 bases of the 1693 base coding sequence including the intron.

### Measurement of pollen exclusion scores

Measurement of pollen exclusion scores was based on the ability of the hybrids to exclude pollen that produced purple kernels in successful pollinations. This pollen can be considered a proxy for unwanted contaminating pollen with the advantage that purple kernels that are easily detected and counted. We refer to pollen that produces blue or yellow grain as blue or yellow pollen, respectively, even though all pollen is yellow in color. Three types of pollinations were carried out to quantify pollen exclusion (Table 1; Supplementary Fig. 1). First, pollen was collected from 4 to 6 blue pollen donor plants, mixed, and tested on *Ga1-s* controls, *ga1* controls and experimental lines. These blue-only pollinations verified that our blue pollen was viable and produced blue kernels on each of the experimental lines and controls used in the study. Complete exclusion is often seen in individual ears of *Ga1-s*/*Ga1-s* lines, but it is rarely complete in the “blue-only” pollinations across replicates. The color of the few kernels that are created with the “blue-only” pollinations informed us that all hybrids in the study supported the development of blue kernels. This control is important because a few hybrids do not support the development of blue color when pollinated with blue pollen. Following the “blue-only” pollinations, *Ga1-s* pollen from 2–3 plants was then added to the remaining blue pollen to create a mix of blue and yellow pollen, and both control pollinations and experimental pollinations were conducted with these “Blue + Ga1-s” mixes. The goal was to have two “blue-only” mixes and at least three different “Blue + Ga1-s” mixes used on every experimental plot, and three control pollinations with each pollen mix. Mixes are labeled “Mix A” through “Mix I” in Supplemental Table 1 so that all ears pollinated from the same mixture could be tracked. The control pollinations allowed for verification of the blue:yellow ratio in each “Blue + Ga1-s” pollen mix. Our target blue:yellow ratio was 9:1 because an excess of blue pollen would allow more sensitivity to detect low amounts of pollen contamination than would be possible with a 1:1 mix ratio. Our control pollinations showed that our pollen mix ratios were all between 9:1 and 9.5:1 (data not shown), so we did not correct for differences in pollen mix ratios when processing the experimental Blue + Ga1-s mix data. Careful notes allowed the “blue-only” mixes to be matched to their corresponding “Blue + Ga1-s” mixes. In this way, each experimental plot was tested by pollinating between five and ten ears with pollen mixes to measure degree of pollen exclusion with the quality of each mix confirmed by control pollinations.

**Table 1:** Three types of pollinations performed in this study.

Designation	Male 1		Male 2		Female		Purpose
	Grain color	Allele	Grain color	Allele	Grain color	Allele	
Blue-only	Blue	ga1/ga1	–	–	Yellow	Ga1-s/Ga1-s	Verify blue function and exclusion
Blue + Ga1-s mix	Blue	ga1/ga1	Yellow	Ga1-S/Ga1-S	Yellow	Ga1-s/Ga1-s	Quantify pollen exclusion
Control mix	Blue	ga1/ga1	Yellow	Ga1-S/Ga1-S	Yellow	ga1/ga1	Establishes ratio of pollen types in mix

Because of variation in blue kernel color development, it is occasionally difficult to distinguish between yellow and blue kernels. To overcome problems with obtaining exact blue and yellow kernel counts from each ear, we assigned a rating to each ear pollinated with a “Blue + Ga1-s” pollen mix based on the percentage of blue kernels present. A rating of 5 had 0–10% blue kernels, a rating of 4 had 10–25% blue kernels, a rating of 3 had 25–50% blue kernels, a rating of 2 had 50–75% blue kernels and a rating of 1 had 75%-100% blue kernels. With this rating system, a higher rating indicates the corn line excludes foreign (non-*Ga1-s*) pollen well. Each ear was rated individually and the ratings of all ears in a plot produced by Blue + Ga1-s pollinations were averaged prior to statistical analysis. Ears produced by control pollinations were processed in the same way (Supplemental Table 1).

### Statistical methods

Because our raw data is classified into scores ranging from 1 to 5, these data violate the assumption of continuous data, however, averaging across 5–10 subsamples taken from each plot resulted in data that closely resembled continuous data. The fit quality of the model as determined by the distribution of the residuals (Supplemental Fig. 2) suggests that this violation had minimal impact on the validity of the statistical analysis.

The experiment was analyzed by fitting the mean pollen exclusion rating for each plot as the response variable in the following linear model:

$${\text{Y}}_{{{\text{ijk}}}} = \, \mu + {\text{Year}}_{{\text{i}}} + {\text{Class}}_{{\text{j}}} + {\text{Genotype}}\left[ {{\text{Class}}} \right]_{{{\text{ijk}}}} + \left( {{\text{Year }} { \times }{\text{ Class}}} \right)_{{{\text{ij}}}} + \varepsilon_{{{\text{ijk}}}}$$

where Y is the response variable measured in each plot, i.e., the mean of the of the subsamples from each plot. µ is the experiment-wide mean pollen exclusion rating. Year_i_ is the effect of the ith year (i = 2019 or 2020). Class_j_ is the effect of the jth class (j = Dent, Pop or PuraMaize). Genotype[Class]_jk_ is the effect of kth genotype nested within the jth class. (Year × Class)_ij_ is the interaction effect of the ith year and the jth class.

All effects were modeled as fixed effects. The model was fit using the standard least squares method. The significance of the effects was determined by an F-test of the ratio of the mean squares of each effect to the mean squares of the error term.

This model explained 93% of the variance in the experiment. We also tested a model containing a fifth effect, the interaction between year and genotype within class (Year x Class[Genotype]_ijk_) and this model explained slightly more of the variance (95%). However, this effect was not significant, so we used the four effect model for the analyses presented. The residuals of this model fit a normal distribution well (Supplementary Fig. 2).

## Results

### Characterization of the *Ga1-s* allele

All popcorn and *Ga1-s* dent corn hybrids used in the study were known to have a pollen excluding phenotype, but none of the pollen excluding alleles present in these hybrids had been characterized molecularly. We PCR amplified the *ZmPme3* gene which is part of the *Ga1-s* allele^13,14^ from each of these hybrids, confirming that they all contained a *Ga1-s* allele. We then sequenced the amplified gene to determine if any of the hybrids contained a different version of the allele. The DNA sequence of all *Ga1-s* lines in the study was the same as the published allele, which was first characterized in White Cloud popcorn. This observation is important because it suggests that observed variation in degree of pollen exclusion is not due to sequence variation in the *ZmPme3* gene.

### Pollen exclusion evaluation

In order to understand the variation in pollen exclusion among hybrids used in this study, we tested the significance of each source of variation introduced by the experimental design (Table 2). The year effect was significant, although it was small, explaining only 1.9% of the total variance. It is important to understand environmental effects on pollen exclusion because they could impact the effectiveness of a pollen exclusion system in commercial production. With only two years in this study, this effect needs to be examined more thoroughly.

**Table 2:** ANOVA of pollen exclusion ratings.

Effect	DF	% Variance explained	Significance^b^	Sums of squares
Year	1	1.9	0.0016**	2.3
Class^a^	2	81.7	< 0.0001**	97.9
Genotype (class)	13	8.6	0.0003**	10.3
Year × class	2	0.3	0.4313 n.s.	0.3
Model total	18	92.5		110.8
Error	45	7.5		9.0
Total	63	100		119.8

^a^Popcorn, PuraMaize (Ga1-s), or dent corn (ga1).

^b^Probability of > F. **, statistically significant at a threshold probability of 0.05. n.s., not statistically significant.

**Figure 1:**
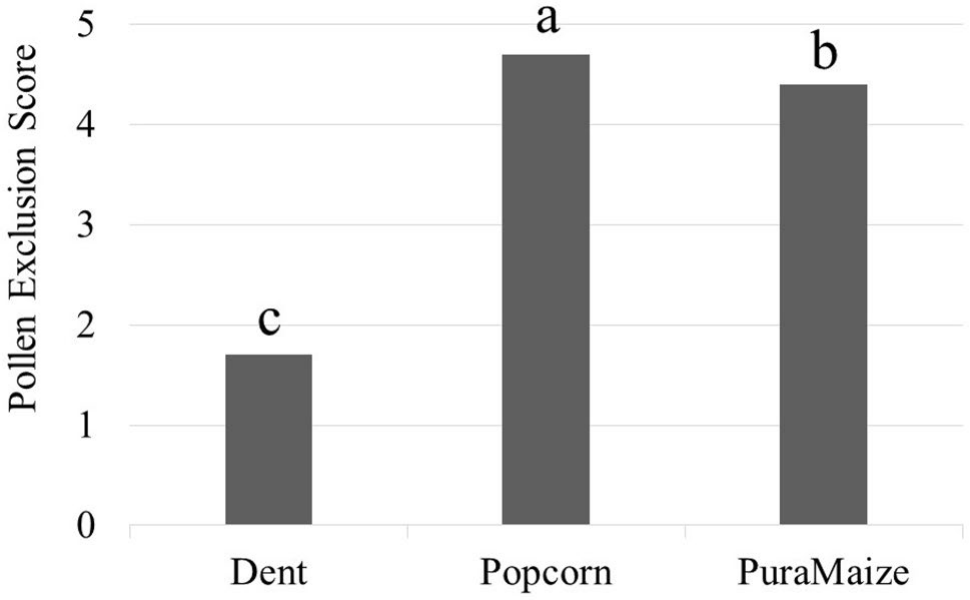
Pollen exclusion ratios of three classes of maize. Classes labelled with different letters are statistically different at p > 0.05.

The majority of the variance (81.7%) was explained by the Class effect (Table 2). Variation observed among the Dent, Popcorn or PuraMaize classes was unlikely to be due to chance. This was expected because the Dent class does not contain a pollen exclusion mechanism while the Popcorn and PuraMaize classes do. Of greater interest is comparison of the mean pollen exclusion value among the three classes (Fig. 1). Each of the three classes were significantly different, with Popcorn having the highest degree of pollen exclusion followed by PuraMaize and Dent corn. The *Ga1*-s allele conferred protection to both Dent and Popcorn, but in different degrees.

The Genotype[Class] effect was significant (Table 2), indicating that variation among genotypes within each class was not likely to be due to chance (Table 3). All of the hybrids containing *Ga1-s* were higher than all of the hybrids without it. However, within the hybrids containing *Ga1-s* there was overlap in exclusion ability among hybrids in the Popcorn and PuraMaize classes. This shows that it is possible to develop PuraMaize hybrids that exclude pollen as well as some popcorn hybrids. This result also suggests that it should be possible to use this method of measuring the degree of pollen exclusion in selection experiments to improve the trait.

**Table 3:** Pollen exclusion ratings of 6 popcorn, 6 PuraMaize and 4 dent corn hybrids.

Hybrid	Significance group^a^	Pollen exclusion rate^b^
Popcorn 1	A	5.0
Popcorn 2	A	5.0
PuraMaize 1	AB	4.9
Popcorn 3	AB	4.8
Popcorn 4	AB	4.7
PuraMaize 2	AB	4.7
PuraMaize 3	AB	4.7
Popcorn 5	ABC	4.5
Popcorn 6	BCD	4.3
PuraMaize 4	BCD	4.2
PuraMaize 5	CD	3.9
PuraMaize 6	D	3.7
Dent corn 1	E	2.6
Dent corn 2	F	1.5
Dent corn 3	F	1.5
Dent corn 4	F	1.2

^a^Hybrids connected by the same letter are not significantly different from each other.

^b^Mean rating, 5 = 0–20% blue kernels, 1 = 80–100% blue kernels.

## Discussion

Because no sequence differences were detected in the *Ga1-s* alleles carried by the dent and popcorn varieties, the most likely explanation for the observed differences in pollen exclusion is the presence of modifier genes that increase or decrease the effectiveness of pollen exclusion. This observation is consistent with Hurst et al., 2019 who mapped loci controlling the degree of pollen exclusion in popcorn lines. Significant variation among *Ga1-s* dent corn hybrids (Table 3) suggest that there may be modifier loci within dent corn as well. This would be an interesting finding since dent corn was developed in the absence of the *Ga1-s* allele in recent years at least. An alternative hypothesis is that modifiers present in *Ga1-s* popcorns were lost during back-crossing leading to significant variation among *Ga1-s* dent corn hybrids. The identification of these modifiers should help to understand the evolution and mechanism of this locus. While we did not detect a statistically significant environmental effect, some of the variation in individual lines may still be attributed to experimental differences that we did not control, so replication and testing in multiple environments is important.

Quantitative measurement of pollen exclusion is extremely difficult, and our method used many controls to account for potential problems with the measurement. We used pollen that produced blue kernels to represent contaminating pollen that would be expected to be excluded by a pollen exclusion system. However, genetic loci such as *C1-I* are known to interfere with the development of blue color from anthocyanin pigments in kernels (reviewed in^15^). We confirmed that each hybrid in the study was capable of developing blue kernels, so presumably the C1-I allele did not impact our pollen exclusion tests. Occasionally we observed a line that excludes very well based on ‘blue-only’ pollinations (i.e., ears produced only a few blue kernels), but produced substantial number of blue kernels from a pollination with a ‘Blue and Ga1’ mix. Our hypothesis is that when pollinating with a large amount of pollen containing both *Ga1-s* and *ga1* pollen at the same time, the *Ga1-s* pollen can overcome the pollen exclusion barrier allowing for the more abundant *ga1* pollen to successfully fertilize the egg. While a mixture of *Ga1-s* and *ga1* pollen could be present in production fields, pollination in production fields occurs over the course of a week rather than all at the same time as it does in our experimental system. Thus, it is unlikely that a *Ga1-s* pollen and multiple *ga1* pollen grains would land on the same silk at the same time, allowing the unwanted *ga1* pollen to fertilize the egg. For this reason, it is likely that our experimental measures of pollen exclusion are lower than the pollen exclusion rates that would be observed in production fields.

While Popcorn and Dent corn had significantly different pollen exclusion rates, both had means > 4 indicating that > 75% of the kernels were yellow in a pollination that heavily favored contaminating blue kernels (> 90% of the kernels were blue in control pollinations with no pollen exclusion system). In actual field conditions, unwanted pollen from other fields would probably be much less abundant than the desirable pollen produced in the same field, again making our experimental system a more stringent assessment of pollen contamination compared to field production systems. Risk analyses may be helpful for identification of an acceptable threshold for the degree of pollen exclusion required to trigger regulatory actions.

## Conclusion

Using a highly sensitive field test for detecting pollen contamination, we learned that both Popcorn and PuraMaize hybrids excluded unwanted pollen significantly better than the *ga1* control hybrids we tested, demonstrating the well-documented effectiveness of the *Ga1-s* allele in regulating cross compatibility and suggesting that the *Ga1-s* allele has good potential for reducing unwanted pollination in either popcorn or dent corn production fields. Popcorn and PuraMaize were not equally effective at excluding unwanted pollen, however. There was significant variation among genotypes in each class and on average, the Popcorn class was significantly better at excluding pollen than the PuraMaize class. While significant, the magnitude of the difference between classes was small. It should be noted that some PuraMaize varieties were better than some popcorn varieties. These data suggest that the presence of *ZmPme3* alone cannot guarantee the pollen exclusion of true *Ga1-s* lines, therefore converting dent corn varieties to *Ga1-s* requires careful testing for their ability to exclude pollen. Until genetic markers for modifiers are identified, this could be achieved by confirmation of complete exclusion by several *ga1* lines. Further, it would be helpful to develop a threshold level of effectiveness of pollen exclusion that could be applied uniformly to both popcorn and dent corn varieties when deciding the level of genetic purity testing necessary for products of maize carrying the *Ga1-s* allele.

## Supplementary Information

Supplementary Information.

## Data Availability

All datasets obtained or studied during this study are incorporated in the manuscript.

## Abbreviations

GMO: Genetically modified organism
USDA: United States Department of Agriculture
