# Supplementary material for: The maize Ga1-s allele confers protection against ga1 pollen in popcorn and dent corn
Source: Sci Rep. 2022 Dec 2;12:20809. doi: 10.1038/s41598-022-25261-8 (PMC9718820; doi:10.1038/s41598-022-25261-8)
Supplement: Supplementary file 1 — Supplementary Information. [file 41598_2022_25261_MOESM1_ESM.pdf]

Supplemental Figure 1. Example of each genotypic class and all four replicates.

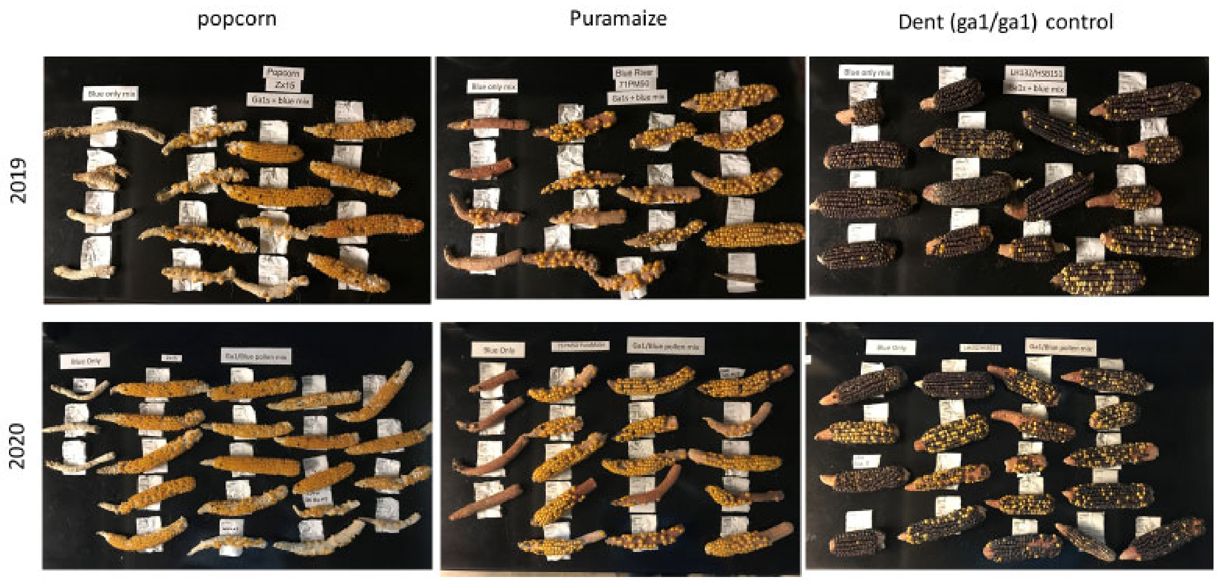

Supplemental Figure 2. Studentized residuals of pollen exclusion measurements plotted as a Normal Quantile Plots. The solid red lines indicate a normal distribution. Dashed Red lines indicate the 95% confidence interval for a normal distribution of the residuals.

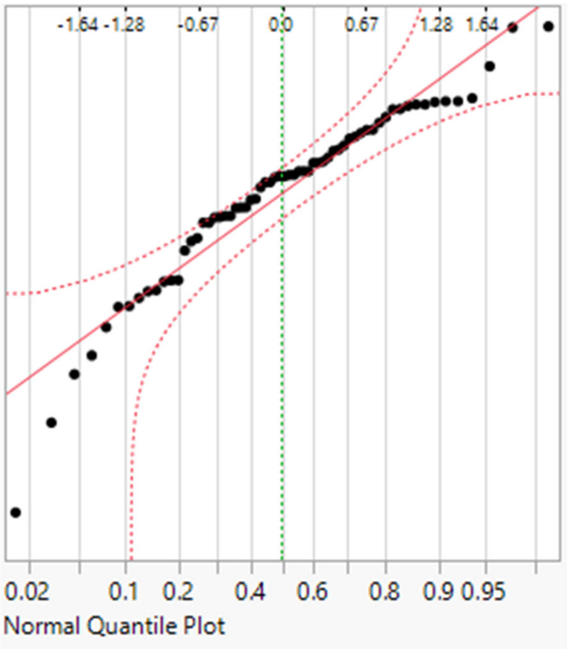

Supplemental Table 1. Individual ear ratings for pollen exclusion as described in Materials and Methods.

Year	Loc Seq#	Rep	Class	Pedigree	Mix A	Mix B	Mix C	Mix D	Mix E	Mix F	Mix G	Mix H	Mix I	Mix average
2019	1049	1	popcorn	1H262	5	5	5	5	5	5				5
2019	1092	2	popcorn	1H262	5	5	5	5	5	5				5
2020	2333	3	popcorn	1H262	5	5	5	5	5	4	5			4.85714286
2020	2367	4	popcorn	1H262	5	5	5	5	5	5	5	5		5
2019	1047	1	popcorn	1H820	5	5	5	5	5	5				5
2019	1068	2	popcorn	1H820	5	5	5	5	5	5				5
2020	2349	3	popcorn	1H820	3	5	4	4	5	4	4	5		4.25
2020	2393	4	popcorn	1H820	5	5	5	4	5	5	4	4		4.625
2019	1052	1	PuraMaize	54PM37 PuraMaize	1	2	2	4	4	2				2.5
2019	1089	2	PuraMaize	54PM37 PuraMaize	4	4	3	4	4	5				4
2020	2331	3	PuraMaize	54PM37 PuraMaize	5	5	5	5	3	3	4	5		4.375
2020	2369	4	PuraMaize	54PM37 PuraMaize	3	5	3	3	5	3	5	5		4
2019	1028	1	PuraMaize	58PM36 PuraMaize	5	5	4	4	5	5				4.66666667
2019	1069	2	PuraMaize	58PM36 PuraMaize	5	4	5	5	5	5				4.83333333
2020	2351	3	PuraMaize	58PM36 PuraMaize	5	5	3	4	4	5	5	5		4.5
2020	2374	4	PuraMaize	58PM36 PuraMaize	4	5	5	5	5	5	5	5		4.875
2019	1030	1	PuraMaize	66PM26 PuraMaize	1	4	4	4	5	3				3.5
2019	1071	2	PuraMaize	66PM26 PuraMaize	5	5	4	4	4	4				4.33333333
2020	2332	3	PuraMaize	66PM26 PuraMaize	5	2	5	5	5	5	4			4.42857143
2020	2390	4	PuraMaize	66PM26 PuraMaize	5	5	5	5	5	4	4	5	4	4.66666667
2019	1054	1	PuraMaize	71PM50 PuraMaize	5	5	5	5	5	5	5			5
2019	1091	2	PuraMaize	71PM50 PuraMaize	5	5	5	5	5	5				5
2020	2354	3	PuraMaize	71PM50 PuraMaize	5	5	5	4	5	4	5	5		4.75
2020	2368	4	PuraMaize	71PM50 PuraMaize	5	5	5	4	4	5	5			4.71428571
2019	1034	1	dent	B104 x CLRQ 00502/B1	2	2	3	1	2	2	3			2
2019	1070	2	dent	B104 x CLRQ 00502/B1	2	2	3	2	3	2				2.33333333
2020	2352	3	dent	B104 x CLRQ 00502/B1	3	3	3	4	3	3	2	2	2	2.875
2020	2392	4	dent	B104 x CLRQ 00502/B1	3	4	4	3	3	3	2	3		3.125
2019	1053	1	dent	B73/Mo17	2	2	1	1	1	1				1.33333333
2019	1067	2	dent	B73/Mo17	1	1	1	1	1	2				1.16666667
2020	2328	3	dent	B73/Mo17	1	1	1	1	1	1	1	1	1	1
2020	2387	4	dent	B73/Mo17	2	3	1	1	1	1	1	1	1	1.33333333
2019	1051	1	dent	BS13(S)C8)/OH 514	2	1	2	1	1	1				1.33333333
2019	1093	2	dent	BS13(S)C8)/OH 514	1	1	1	2	1	2				1.33333333
2020	2350	3	dent	BS13(S)C8)/OH 514	2	2	1	1	1	2	1	1		1.375
2020	2373	4	dent	BS13(S)C8)/OH 514	2	1	3	2	1	2	2	3		2
2019	1050	1	PuraMaize	HPM603 PuraMaize	5	5	4	5	4	5				4.66666667
2019	1087	2	PuraMaize	HPM603 PuraMaize	5	3	4	5	4	3				4
2020	2329	3	PuraMaize	HPM603 PuraMaize	5	5	5	5	5	5	5	5	5	5
2020	2371	4	PuraMaize	HPM603 PuraMaize	5	5	5	5	5	5	5	5	5	5
2019	1033	1	dent	LH132/HSB151	2	1	1	1	1	1				1.16666667
2019	1094	2	dent	LH132/HSB151	1	1	1	1	1	2				1.16666667
2020	2334	3	dent	LH132/HSB151	1	3	2	2	2	2	3	1		2
2020	2394	4	dent	LH132/HSB151	3	3	1	2	1	1	1			1.71428571
2019	1029	1	PuraMaize	QM3922 PuraMaize	5	5	5	4	4	4	4	4		4.375
2019	1088	2	PuraMaize	QM3922 PuraMaize	3	3	1	2	2	3				2.33333333
2020	2348	3	PuraMaize	QM3922 PuraMaize	5	5	4	5	5	5	5	5	5	4.88888889
2020	2372	4	PuraMaize	QM3922 PuraMaize	5	4	5	4	3	4	5	3		4.125
2019	1031	1	popcorn	Zx15	5	5	5	5	5	5				5
2019	1090	2	popcorn	Zx15	5	5	5	5	5	5				5
2020	2347	3	popcorn	Zx15	5	5	5	5	5	5	5	5		5
2020	2370	4	popcorn	Zx15	5	5	5	5	5	5	5	5		5
2019	1027	1	popcorn	Zx2	5	4	5	5	5	5				4.83333333
2019	1073	2	popcorn	Zx2	4	4	4	5	5	5				4.5
2020	2353	3	popcorn	Zx2	5	5	5	5	5	5	5	5		5
2020	2389	4	popcorn	Zx2	5	5	5	5	5	5	5	5	5	5
2019	1032	1	popcorn	Zx40	5	5	4	5	5	4	5			4.66666667
2019	1074	2	popcorn	Zx40	5	3	4	3	3	4				3.66666667
2020	2330	3	popcorn	Zx40	5	5	5	5	5	5	5	5	5	5
2020	2391	4	popcorn	Zx40	3	5	5	5	5	5	5	5		4.75
2019	1048	1	popcorn	Zx44	5	3	2	5	3	3				3.5
2019	1072	2	popcorn	Zx44	5	4	4	5	5	3				4.33333333
2020	2327	3	popcorn	Zx44	3	5	5	5	4	5	5	5	5	4.66666667
2020	2388	4	popcorn	Zx44	5	5	4	5	4					4.6
